# The impact of natural and urban environmental settings on exercise and perceptual responses during virtual reality-based exercise

**DOI:** 10.3389/fpsyg.2026.1758056

**Published:** 2026-03-17

**Authors:** Emanuel Festino, Olga Papale, Cristina Cortis, Andrea Fusco, Angela Hibbs, Mohammed Khudair, Kandianos E. Sakalidis, Gill Barry, Florentina J. Hettinga, Gavin D. Tempest

**Affiliations:** 1 Department of Human Sciences, Society and Health, University of Cassino and Lazio Meridionale, Cassino, Italy; 2 European University of Technology, Cassino, Italy; 3 Department of Medicine and Aging Sciences, University “G. d’Annunzio” of Chieti-Pescara, Chieti, Italy; 4 School of Sport, Exercise and Rehabilitation, Faculty of Health and Wellbeing, Northumbria University, Newcastle upon Tyne, United Kingdom; 5 School of Psychology, Cardiff University, Cardiff, United Kingdom; 6 School of Psychology, Faculty of Health and Wellbeing, Northumbria University, Newcastle upon Tyne, United Kingdom; 7 Department of Human Movement Sciences, Faculty of Behavioral and Human Movement Sciences, Vrije Universiteit, Amsterdam, Netherlands

**Keywords:** cycling, engagement, enjoyment, flow, virtual exercise

## Abstract

**Introduction:**

Exercise in virtual reality (VR) is engaging and provides a positive experience, contributing to long-term adherence. Psychological responses such as flow, a state of optimal engagement, and enjoyment, may contribute to these benefits. However, it is unclear whether cycling exercise in different virtual environments influence exercise and perceptual responses. Therefore, this study aimed to evaluate the effects of a natural and urban non-immersive VR environment versus no VR on exercise and perceptual responses during cycling activity.

**Methods:**

Twenty-three physically active young adults completed 10 min of self-paced indoor cycling in three randomized conditions: No VR, and VR with Nature (Nature VR) and Urban (Urban VR) scenes. Power output, speed, heart rate, and Rating of Perceived Exertion were recorded. After each condition, participants completed the Flow State Scale (FSS) and Physical Activity Enjoyment Scale (PACES). Repeated-measures analysis of variance (ANOVA) was used to evaluate exercise and perceptual responses across conditions. Statistical significance was set at *p* < 0.05.

**Results:**

No significant differences were found between conditions for exercise variables. The Nature VR condition reached higher values for Action-Awareness Merging (4.19 ± 0.64), Loss of Self-Consciousness (4.46 ± 0.54), and Unambiguous Feedback (3.85 ± 0.66) compared with No VR (Action-Awareness Merging = 3.59 ± 0.90; Loss of Self-Consciousness = 3.76 ± 1.00; and Unambiguous Feedback = 3.25 ± 0.64). Both VR conditions showed significant differences in Autotelic Experience (Nature = 3.92 ± 0.61; Urban = 3.76 ± 0.59) and PACES (Nature = 30.34 ± 3.88; Urban = 29.08 ± 4.32) compared to No VR (Autotelic Experience = 3.04 ± 0.84; PACES = 25.34 ± 4.40).

**Discussion:**

Nature VR provided additional benefits on specific flow dimensions compared with No VR cycling. These findings support the use of non-immersive VR, particularly natural scenes, as a strategy to improve the exercise experience, potentially supporting exercise adherence.

## 1 Introduction

Physical activity is beneficial for physical and mental health, regardless of age, gender, and health status (Bull et al., 2020). Although the benefits of physical activity and exercise are numerous and widely recognized, the number of sedentary/low-active people worldwide continues to grow every year (Strain et al., 2024). A possible explanation for data of this magnitude could be attributed to the fact that sometimes people find exercise boring or unenjoyable (Wolff et al., 2021). Compared with indoor exercise, outdoor activity in nature is associated with greater enjoyment and a more positive subjective experience (Gladwell et al., 2013; Papale et al., 2026), likely due to the serene visual characteristics such as green colors, vegetation, trees, and landscape features, which provide a nice distraction, improving mood, and reducing the perception of effort (Gladwell et al., 2013). Distraction during exercise may also arise from exposure to novel or unfamiliar environments, including urban settings, suggesting that both stimuli may contribute to perceptual responses. However, urban environments provide a higher perceptual and cognitive load than natural environments, due to the presence of multiple competing visual natural and urban scenes (i.e., signs and vehicles) (White and Shah, 2019; Fan et al., 2025). The increased cognitive load in an urban environment could introduce psychological strain and attentional fatigue (White and Shah, 2019; Fan et al., 2025). Green or natural spaces are advantageous for health, increasing mental well-being and markers of physiological health (Gladwell et al., 2013; Papale et al., 2023; Papale et al., 2026). However, although outdoor exercise has mental and physical health benefits, it is not always accessible or feasible. In fact, environmental factors like weather conditions, pollution, and safety concerns can limit opportunities for outdoor exercise, highlighting the need for innovative solutions to enhance indoor activities (e.g., make them more enjoyable) (Pagan et al., 2003; Alghamdi et al., 2023; Seo et al., 2023). Virtual reality (VR) training programs offer the opportunity to enhance the indoor exercise experience (Qian et al., 2020; Zhou, 2020; Hibbs et al., 2024; Colombo et al., 2025). VR is operationally defined as a digital technology in which sensory experiences are artificially created, prompting users to manipulate objects within an artificial environment (Wu et al., 2023).

Previous literature (Lind et al., 2009) has highlighted that attentional focus and cognitive distraction during exercise can modulate perceived exertion and affective responses, influencing exercise adherence. In this context, cognitive strategies could shift attention away from internal discomfort, by dissociation, thereby enhancing the exercise experience (e.g., exercise may feel less effortful and increase or maintain more positive affect), particularly during submaximal exercise (Lind et al., 2009). Exercise in VR is a cognitive distraction that has the potential to promote physical activity and health behaviors, overcoming many barriers, such as environmental factors (e.g., going outdoors in poor weather) (Zhou, 2020; Colombo et al., 2025). The VR environment offers the experience of being in different indoor and outdoor environments (such as urban, country or coastal surroundings) whilst at home (Zhou, 2020). Immersive VR uses head-mounted displays, body movement sensors, real-time graphics, and/or advanced interface devices to simulate a complete virtual environment for users, whereas non-immersive VR utilizes an interface, such as a screen TV/computer screen (Qian et al., 2020).

VR technology has been used in the management of neurological and psychiatric disorders, to promote physical activity and health promotion among healthy low-active populations (Bird et al., 2019; Qian et al., 2020; Wu et al., 2023; Mocco et al., 2024). Studies have shown that through the use of VR during exercise, mental factors such as enjoyment, affective valence, and flow experience can be positively influenced, potentially increasing exercise adherence in the long term (Liu et al., 2019; Mouatt et al., 2023; Mocco et al., 2024; Palmieri and Deutsch, 2024; Polechoński et al., 2024; Colombo et al., 2025). Exercise enjoyment is defined as a positive response to the movement experience that reflects feelings such as joy, and fun derived from the activity (Raedeke, 2007), and affective valence refers to the degree of pleasure or displeasure experienced during exercise (Bird et al., 2019). Flow experience has a broader meaning referring to an intrinsically rewarding state where individuals feel fully absorbed in an activity, experiencing control and ease, even during challenge (Jackson and Marsh, 1996). Although immersive VR has been shown to provide higher enjoyment, positive mood states, motivation and flow experience during exercise than non-immersive VR (Hibbs et al., 2024; Liu et al., 2024; Polechoński et al., 2024; Colombo et al., 2025), immersive VR has several challenging issues, such as expensive equipment and lack of infrastructure support (Burdea, 2003). Instead, non-immersive VR, through the use of a monitor or screen, represents an accessible and low-cost strategy to achieve similar psychological benefits of VR exercise.

Despite the well documented psychological and physiological benefits of VR exercise, it is important to examine which types of VR scenario or environment provides the optimal experience during indoor exercise. In outdoor contexts, natural environments, such as coastal regions, parks, and forests, are often associated with lower stress, improved mood, and enhanced cognitive function compared to city or urban areas (Gladwell et al., 2013; Bayrakdar and Güneş, 2025). Furthermore, walking in a natural environment has been shown to promote a more positive experience (such as lower tension and increased calmness) than walking in an urban environment (Kinnafick and Thøgersen-Ntoumani, 2014). In the same study, pleasure and activation (arousal) were highest when walking in the natural environment compared to sitting in the urban environment, suggesting that being physically active in nature may acutely enhance affective and experiential responses during exercise (Kinnafick and Thøgersen-Ntoumani, 2014).

VR offers dissociation from internal signals associated with exercise (such as increases in heart rate and effort), whereby “cognitive distraction” and increased immersion in the task is a proposed mechanism for the improved psychological responses observed (Bird et al., 2019). Being outdoors leads to a connectedness with nature and visual recognition of characteristic features such as the color green, and geometrical fractals is beneficial for health (Joye and van den Berg, 2011; Akers et al., 2012; Cervinka et al., 2012; Lahart et al., 2019). Therefore, incorporating natural scenes during indoor cycling using VR may be an effective way to improve the experience of this activity indoors. Studies have indicated that the complexity of urban conditions may overwhelm one’s attentional capacity, and in turn, drain attention and lead to a less positive experience (Kaplan and Kaplan, 2011; Kinnafick and Thøgersen-Ntoumani, 2014), hindering restoration (i.e., the process of recovery from stress, fatigue) associated with natural environments. Given the influence of environmental cues (such as natural and urban scenes) on psychological responses, it is important to further understand these effects using VR during indoor exercise. Investigating how VR-based presentation of natural and urban scenes influences exercise and perceptual responses to exercise may help to identify which type of environment may provide greater psychological benefit and a more positive exercise experience. This knowledge could be useful for optimizing the use of VR to enhance motivation and long-term adherence to exercise. Therefore, the aim of this study was to evaluate the effects of different virtual environments (natural *vs.* urban) and a traditional non-VR cycling condition on exercise and perceptual responses during cycling activity. It was hypothesized that both VR conditions could enhance exercise responses and psychological factors, such as flow experience and enjoyment, compared with traditional non-VR cycling. Furthermore, it was expected that natural scenes would lead to a more positive exercise experience compared to urban scenes.

## 2 Materials and methods

### 2.1 Study design

The study used a within-subjects design with three conditions: (A) No VR condition, where participants cycled in front of a blank screen; (B) Nature VR condition, where participants cycled while viewing a projected natural environment; and (C) Urban VR condition, where participants cycled while viewing a projected urban environment.

A non-immersive VR setup, consisting of a large screen monitor, displaying the corresponding virtual scenarios (natural or urban), positioned in front of the participant, was used. The exposure to each condition was randomized to minimize order effects such as learning, fatigue, or familiarity with the task.

### 2.2 Participants

Twenty-three healthy young adults (6 females, 17 males, age: 24.13 ± 6.04 years) from the student population of the Northumbria University, volunteered to participate in the study. This population was selected due to their homogeneity in terms of age, general health status, and physical activity level, to minimize potential confounding variables (Liu et al., 2019; Colombo et al., 2025). Participants were excluded if they reported pre-existing conditions such as neurological conditions, cardiovascular, respiratory, and/or metabolic diseases, hypertension, osteoporosis, musculoskeletal injury of the back or lower extremities occurring during the past year, visual and vestibular disorders. Participants reported no prior engagement or extensive use of VR-based exercise. All participants reported meeting 150 min of physical activity per week, evaluated through the International Physical Activity Questionnaire (Craig et al., 2003).

The study was conducted according to the guidelines of the 1964 Declaration of Helsinki and it was reviewed and approved by the Ethics Committee at Northumbria University (submission reference number: 7930). All participants took part in the study voluntarily, provided written informed consent, and were informed of their right to withdraw from the study at any time and for any reason.

### 2.3 Procedures

Participants completed three laboratory visits (at least 24 h apart): a familiarization visit, one experimental visit including the No VR condition, and a second experimental visit including non-immersive VR scenarios (Nature *vs.* Urban). During the familiarization session, participants were introduced to the bike and screen monitor setup. The anthropometric characteristics and physical activity levels of the participants were assessed. Body weight and height were determined using a precision instrument that combines a scale and stadiometer accurate to 0.1 kg and 0.1 cm (Seca, model 709, Vogel and Halke, Hamburg, Germany) (Table 1). Additionally, the body mass index was calculated.

**Table 1:** Means and standard deviations of anthropometric characteristics of participants.

Variable	Male	Female	Total
Age (years)	24.23 ± 6.31	23.83 ± 5.74	24.13 ± 6.04
Height (m)	1.81 ± 0.07	1.64 ± 0.05	1.77 ± 0.10
Weight (kg)	83.45 ± 14.28	69.53 ± 17.72	79.82 ± 16.09
Body mass index (kg/m^2^)	25.19 ± 3.11	25.86 ± 7.86	25.37 ± 4.60

**Figure 1:**
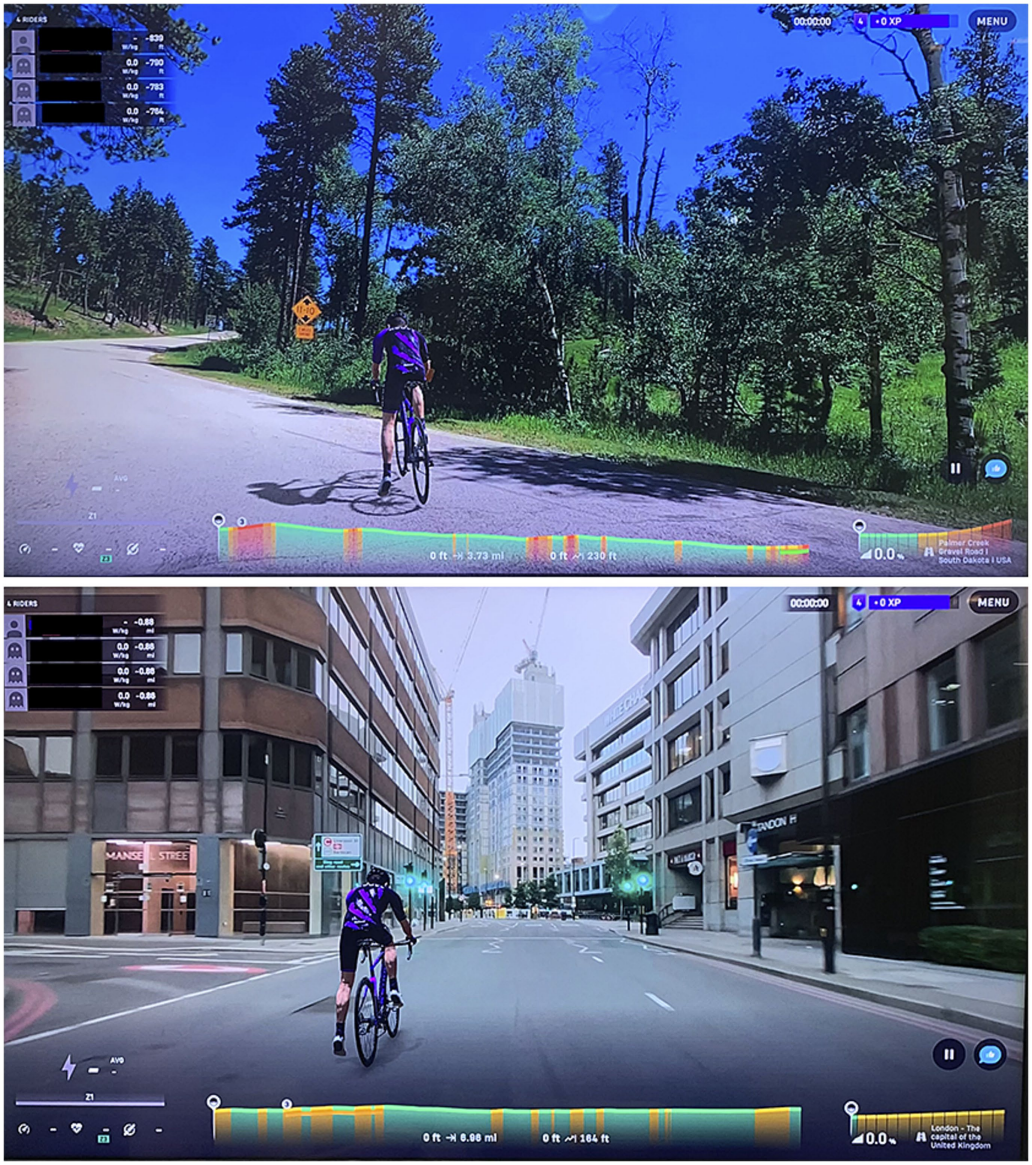
Representation of the non-immersive virtual environments used in the study. Top panel: Nature virtual reality condition displaying the Palmer Creek Gravel Road. Bottom panel: Urban virtual reality condition depicting London cityscape.

During familiarization, the maximum heart rate of the participants was estimated through the 220 – age equations (Camarda et al., 2008). During all conditions, the cycling exercise was performed on the Cyclus 2 bicycle ergometer (Cyclus 2^®^, RBM elektronik-automation GmbH, Leipzig, Germany). The height of the ergometer seat and of the screen monitor were adjusted to fit with the individual anthropometric characteristics. Participants were given instructions on the experimental procedure, and questionnaires were administered at the end of each condition were introduced. The screen monitor was a 50-inch LED display (NEC MultiSync E507Q, NEC Corp., Tokyo, Japan) with 4K Ultra High-Definition resolution (3,840 × 2,160 pixels). The monitor was positioned approximately 2 m in front of the participant at eye level, to minimize head and eye movements. The non-immersive VR platform was administered via the Rouvy application (VirtualTraining, Vimperk, and Czech Republic), installed on a MacBook Air (model A1466) and connected to the screen monitor. The application combines indoor cycling with real-route videos, allowing users to ride on authentic paths around the world. The Rouvy software was synchronized with the Cyclus2 bicycle ergometer, allowing the virtual route to advance according to the participant’s pedaling cadence and power. Rouvy offers an interactive experience where cyclists can choose from various video-recorded routes, which are adjusted to simulate the actual terrain conditions (Pereira and Matos, 2024). This immersion type makes workouts more engaging and helps athletes maintain motivation while providing accurate performance metrics (Pereira and Matos, 2024). For the Nature VR condition, the selected route was Palmer Creek Gravel Road (South Dakota, United States), representing a natural, forested environment. For the Urban VR condition, a London cityscape with roads, buildings, and traffic elements was utilized. These routes, in addition to representing two completely different environments, were selected because they were among the highest-rated scenarios within the Rouvy platform’s Cities and Forest categories. A United Kingdom city was also employed for familiarity. The Nature and Urban VR conditions were both completed on the same day. A 30-min recovery interval was provided between the two trials. This recovery period was necessary to allow participants’ heart rate to return to within ±10 beats per min of their resting values, to ensure adequate recovery and minimize the fatigue between conditions. Figure 1 shows the representation of the non-immersive virtual environments used in the study.

The cycling exercise protocol was identical across each of the three conditions. Participants sat on the ergometer for 5 min to measure resting heart rate. This was followed by a 5-min warm-up corresponding to “light” exercise [in line with Borg 6–20 Rating of Perceived Exertion (RPE) scale, (Kilpatrick et al., 2020)]. After the warm-up, participants cycled for 10 min at a self-paced intensity, during which they were instructed to maintain a comfortable but steady effort that they could sustain throughout the trial. In all conditions, they were free to adjust the gears and cadence throughout the session, allowing them to self-regulate resistance and power output. This self-regulated approach was chosen to simulate a natural pacing strategy and to minimize the influence of externally imposed workloads. Immediately after the exercise phase, participants completed a 5-min cool down corresponding to a “light” intensity using the RPE scale.

### 2.4 Exercise and perceptual measurements

#### 2.4.1 Power output, speed, heart rate

Power output (Watt) and speed (km·h^−1^) were continuously collected at a sampling rate of 2 Hz. Throughout all sessions, heart rate was continuously recorded using a chest strap heart rate monitor (Polar H9, Polar Electro Oy, Kempele, Finland) (Hernández-Vicente et al., 2021) connected to the Cyclus2 ergometer. All measurements were averaged across the trial for analysis.

#### 2.4.2 Perceived exertion

Perceived exertion was assessed using the Borg 6–20 RPE Scale, with 6 representing “No exertion at all” and 20 representing “Maximal exertion” (Borg, 1998). Participants were asked every 2 min during the cycling exercise to verbally report their perceived exertion using the Borg 6–20 RPE Scale. For statistical analysis, RPE values were averaged across the 10-min self-paced exercise.

#### 2.4.3 Flow State Scale

To evaluate the exercise experience, participants completed the Flow State Scale (FSS). The FSS is a 5-point Likert scale and consists of 36 items covering nine dimensions of flow, with 1 representing “Strongly disagree” and 5 representing “Strongly agree” (Jackson and Marsh, 1996). FSS assesses nine dimensions of flow: Challenge-Skill Balance, Action-Awareness Merging, Clear Goals, Unambiguous Feedback, and Concentration on Task at Hand, Sense of Control, Loss of Self-Consciousness, Transformation of Time, and Autotelic Experience. After each exercise condition, participants completed the FSS. High scores on each of the nine dimensions signify whether athletes experienced flow. FSS demonstrates good reliability, with Cronbach’s alpha coefficients ranging between 0.80 and 0.87 across its nine subscales (Jackson and Marsh, 1996).

#### 2.4.4 Physical Activity Enjoyment Scale

After completing each exercise condition, participants completed the Physical Activity Enjoyment Scale (PACES) questionnaire to measure their perceived enjoyment. This questionnaire comprises 5 items evaluated on a 7-point Likert scale. For items 1 and 4, the score ranges from 1 (completely agree) to 7 (completely disagree), while for items 2, 3, and 5, the score ranges from 1 (completely disagree) to 7 (completely agree) (Kendzierski and DeCarlo, 1991; Papale et al., 2025). A higher PACES score reflects a greater level of enjoyment. The PACES has been found to have both reliability and validity in physical activity environments, where Cronbach’s alpha coefficients were higher than 0.70 (Graves et al., 2010).

**Figure 2:**
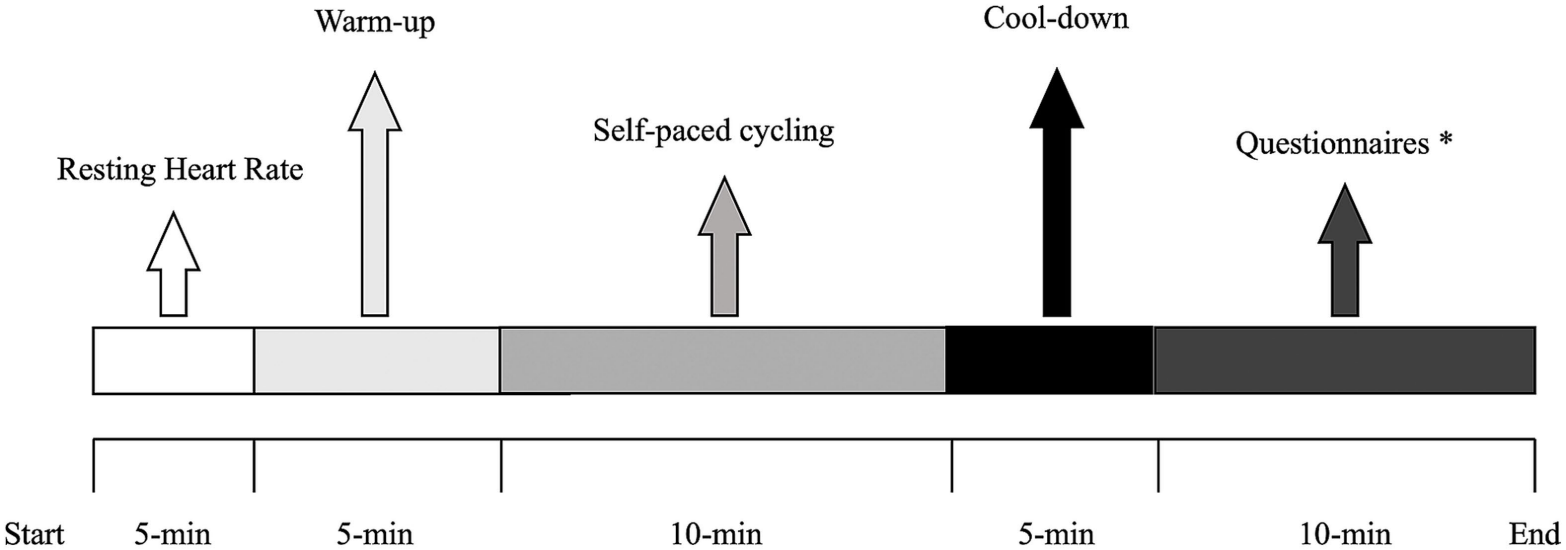
Timeline of the measurements carried out during the three experimental conditions. *Flow State Scale, Physical Activity Enjoyment Scale.

The timeline of the procedures is shown in Figure 2.

### 2.5 Data analysis

The statistical analysis was performed using IBM SPSS Statistics, version 29.0 (IBM Corp, Armonk, NY, United States). Variables were expressed as mean ± standard deviation. Shapiro–Wilk test was used to assess the normal distribution of the data. A repeated-measures analysis of variance (ANOVA) was conducted to compare the three conditions (No VR, Nature VR and Urban VR), as the within-subjects factor, for exercise (power output, speed and heart rate) and perceptual responses (perceived exertion, the nine dimensions of the FSS, and enjoyment). The assumption of sphericity was assessed with Mauchly’s test, and when violated, Greenhouse–Geisser corrections were applied to adjust the degrees of freedom. When significant main effects were found, a Bonferroni correction was applied to follow-up tests to establish significant differences between means. Effect sizes were expressed as Cohen’s *d* whereby an effect size value <0.20 was considered trivial; values between 0.20 and 0.60 were considered small; values that ranged between 0.61 and 1.20 were considered moderate; values between 1.21 and 2.0 were considered large, and >2.0 as very large (Hopkins et al., 2009). Moreover, the 95% Confidence Interval (CI) and Standard Error (SE) were calculated. The level of significance was set at *p* < 0.05. For the non-normally distributed data, the nonparametric Friedman test was applied, followed by Bonferroni-adjusted Wilcoxon signed-rank tests for post-hoc comparisons.

## 3 Results

Means and standard deviations for the exercise and perceptual variables during No VR, Nature VR and Urban VR conditions are shown in Table 2.

**Table 2:** Means and standard deviations of the exercise and perceptual responses variable during No virtual reality, Nature virtual reality, and Urban virtual reality conditions.

Variable	No virtual reality	Nature virtual reality	Urban virtual reality
Power output (Watt)	101.73 ± 52.14	106.95 ± 38.44	99.94 ± 37.98
Speed (Km/h)	25.56 ± 5.49	26.60 ± 3.92	25.85 ± 3.94
Heart rate (bpm)	122.41 ± 21.92	126.06 ± 16.71	123.01 ± 17.63
Percentage of maximum heart rate (%)	62.53 ± 11.17	64.39 ± 8.62	62.85 ± 11.17
Rating of Perceived Exertion	10.83 ± 2.22	10.68 ± 2.14	10.59 ± 2.32
Physical Activity Enjoyment Scale	25.34 ± 4.40^#^	30.34 ± 3.88	29.09 ± 4.32

^#^Indicate significantly lower values in No virtual reality than Nature virtual reality and Urban virtual reality conditions.

### 3.1 Exercise responses

The repeated measures ANOVA showed no significant main effects of VR condition for power output [*F*_(2,44)_ = 0.53; *p* = 0.59], speed [*F*_(2,44)_ = 1.13; *p* = 0.33], or heart rate [*F*_(2,44)_ = 0.57; *p* = 0.57]. During the No VR condition, participants exercised at 62.53 ± 11.17% of age-predicted maximum heart rate. In the Nature VR and Urban VR conditions, the average intensity corresponded to 64.39 ± 8.62% and 62.85 ± 11.17% of age-predicted maximum heart rate, respectively.

### 3.2 Perceptual responses

The repeated-measures ANOVA for RPE, no significant main effects of VR condition [*F*_(2,44)_ = 0.20; *p* = 0.82].

Regarding the FSS dimensions (Table 3), the repeated measures ANOVA showed a significant main effect of VR condition for Action-Awareness Merging [*F*_(2,44)_ = 5.98; *p* = 0.005], Clear Goals [*F*_(2,44)_ = 3.46; *p* = 0.04], and Autotelic Experience [*F*_(2,44)_ = 12.76; *p* < 0.001]. No significant main effects of VR condition were found for Challenge-Skill Balance (*p* = 0.485), Concentration on Task at Hand (*p* = 0.063), and Transformation of Time (*p* = 0.314).

**Table 3:** Means and standard deviations of the Flow State Scale dimensions during No virtual reality, Nature virtual reality, and Urban virtual reality conditions.

Variable	No virtual reality	Nature virtual reality	Urban virtual reality
Challenge-Skill Balance	3.55 ± 0.80	3.71 ± 0.94	3.78 ± 0.54
Action-Awareness Merging	3.59 ± 0.90*	4.19 ± 0.64	3.91 ± 0.54
Clear Goals	3.41 ± 0.83	3.90 ± 0.83	3.60 ± 0.69
Unambiguous Feedback	3.25 ± 0.64*	3.85 ± 0.66	3.61 ± 0.55
Concentration on Task at Hand	3.02 ± 0.96	3.55 ± 0.98	3.25 ± 0.90
Sense of Control	3.70 ± 0.90	4.18 ± 0.72	3.90 ± 0.55
Loss of Self-Consciousness	3.76 ± 1.00*	4.46 ± 0.54	4.11 ± 0.71
Transformation of Time	2.56 ± 0.75	2.79 ± 1.14	2.92 ± 0.97
Autotelic Experience	3.04 ± 0.84^#^	3.92 ± 0.61	3.76 ± 0.59

^*^Indicate significantly lower values in No virtual reality than Nature virtual reality condition. ^#^Indicate significantly lower values in No virtual reality than Nature virtual reality and Urban virtual reality conditions.

After pairwise comparison (with Bonferroni correction), Action-Awareness Merging showed higher scores in the Nature VR compared to No VR condition only (*p* = 0.026; 95% CI = 0.061 to 1.135; SE = 0.207; *d* = 0.6). Clear Goal showed no significant differences between conditions (all *p* > 0.05); and Autotelic Experience, showed lower scores in the No VR condition compared to Nature VR (*p* < 0.001; 95% CI = −1.407 to −0.354; SE = 0.203; *d* = 0.9) and Urban VR (*p* = 0.002; 95% CI = −1.182 to −0.252; SE = 0.179; *d* = 0.83).

**Figure 3:**
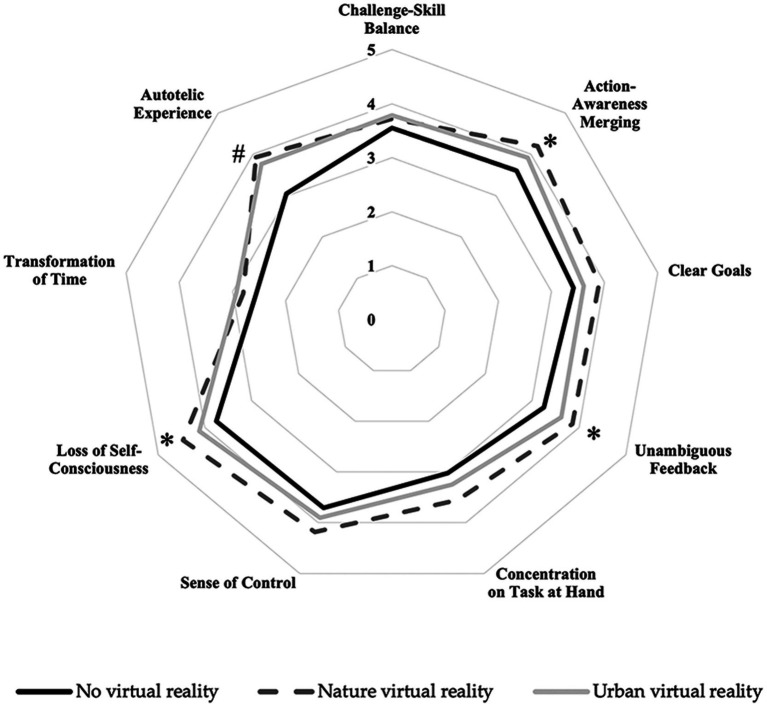
Radar chart of Flow State Scale dimensions across the three cycling conditions. The solid black line represents the No virtual reality, the dark gray dashed line represents the Nature virtual reality, and the light gray solid line represents the Urban virtual reality. *Indicate significantly lower values in No virtual reality than Nature virtual reality condition; ^#^Indicate significantly lower values in No virtual reality than Nature virtual reality and Urban virtual reality conditions.

The Friedman test revealed no significant main effect of VR condition for Sense of Control [*χ*^2^_(2)_ = 4.12; *p* = 0.127]. Conversely, a significant main effect for Loss of Self-Consciousness [*χ*^2^_(2)_ = 12.03; *p* = 0.002] and Unambiguous Feedback [*χ*^2^_(2)_ = 9.237; *p* = 0.01] was shown. Pairwise comparisons with Bonferroni correction showed higher scores for Loss of Self-Consciousness in the Nature VR compared to the No VR condition only (*p* = 0.008; *d* = 0.73), and Unambiguous Feedback was higher in the Nature VR than the No VR condition only (*p* = 0.024; *d* = 0.71). Figure 3 shows the graphical representation of the flow dimensions during the three conditions.

For PACES, repeated-measures ANOVA showed a main effect for VR condition [*F*_(2,44)_ = 9.89; *p* < 0.001]. Bonferroni correction showed that enjoyment was lower in the No VR condition than Nature VR (*p* = 0.002; 95% CI = −8.201 to −1.799; *d* = 0.84) and Urban VR (*p* = 0.30; 95% CI = −7.173 to −0.306; *d* = 0.59).

## 4 Discussion

The study aimed to evaluate the effects of different virtual environments (natural vs. urban) compared to a No VR condition on exercise and perceptual responses during cycling activity. In partial support of our hypothesis, while differences were not found in exercise responses, the different projected virtual environments could play a key role in influencing users’ experiential responses, especially when people use a natural setting.

Following American College of Sports Medicine exercise prescription guidelines (Garber et al., 2011), the intensity reached during each condition, in terms of percentage of maximum heart rate and RPE, was between “light” (57%–63% of maximum heart rate; 9–11 RPE) and “moderate” (64%–76% of maximum heart rate; 12–13 RPE). Cycling in VR (regardless of the type of scenario) did not influence exercise responses. This could be due to the exercise protocol, being self-paced, allowing participants to regulate their effort and maintain a preferred level of exertion, regardless of using the VR. In fact, literature (Runswick, 2023) showed that when cycling protocols allow the participant to control the workload, individuals perceive the same level of effort, whether they tend to work harder or longer. Instead, when the work rate is controlled by operators (not self-paced), different results were found. Several studies showed that when the workload is controlled, participants rated their RPE lower when performing an exercise in VR than when performing without feedback (Matsangidou et al., 2019; Finnegan et al., 2023; Colombo et al., 2025). Another reason is the lack of a competitive aspect. In fact, previous research has reported greater exercise intensity in VR cycling with competitive challenges than non-competitive, indicating that performance-oriented tasks may be necessary to observe condition effects on physiological outputs (Palmieri and Deutsch, 2024, 2025). This suggests that, during self-paced exercise, participants can manage their effort to maintain a comfortable and familiar level of exertion, regardless of VR environmental manipulation.

Although participants worked at the same intensity, significant differences in perceptual responses were observed. A significant main effect of VR condition on specific dimensions of the FSS and enjoyment indicated higher levels of enjoyment and exercise experience during VR compared to No VR. The feeling of pleasure during exercise is an important factor in physical activity participation and compliance (Costa et al., 2015). This is based on the hedonic theory of motivation (Kaczmarek, 2017), which suggests that when exercise is performed by the participant and is perceived as pleasurable, it will likely be repeated (Costa et al., 2015). The nature VR scene was shown to improve the exercise experience (as measured using the FSS). Despite no significant differences between the Nature VR and Urban VR conditions, during a natural scene, participants reported higher scores for Action-Awareness Merging, Loss of Self-Consciousness, Unambiguous Feedback, and Autotelic Experience dimensions in comparison to No VR. These dimensions suggest that when participants cycle in a ‘green’ environment through VR, they may experience a stronger sense of immersion and clearer feedback about the activity, with reduced awareness of self as separate from the actions being performed. Immersion in a natural scene may have reduced participants’ self-monitoring and performance-related concerns, developing a state of effortless engagement where actions feel instinctive and confident. These results are in line with previous work (Hibbs et al., 2024) that highlighted the benefit of VR, especially non-immersive setups, in the users’ satisfaction during cycling exercise, by increasing the engagement during activity, experiencing a high level of focus, control, and pleasure. While a previous study (Polechoński et al., 2024) reported greater benefits for immersive VR, the present findings suggest that non-immersive VR may also provide positive perceptual responses under certain conditions, such as self-paced exercise and visually engaging scenarios. However, in the present study, only the Autotelic Experience dimension achieved higher scores in both natural and urban scenes than in traditional indoor cycling, suggesting that VR, regardless of the environment, can make the experience more intrinsically rewarding.

Although no differences in flow dimensions were found between the two VR conditions, for the other three flow dimensions (Action-Awareness Merging, Unambiguous Feedback, Loss of Self-Consciousness) only the Nature VR showed higher scores than the No VR condition. For example, participants who performed in the nature scenario had a deeper sense of engagement in the activity compared to those cycling in the No VR condition, whereas the Urban VR condition did not differ from the traditional setup. Different studies have shown that the benefits of physical activity in natural environments may confer additional mental health benefits compared to those resulting from equivalent activity in an urban/built, or indoor environment (Bowler et al., 2010; Lahart et al., 2019; Papale et al., 2025). This is associated with the concept that exposure to natural scenes can produce positive psychological states, such as increased positive affect, enjoyment, or satisfaction, and a reduction in psychophysiological stress (Bowler et al., 2010; Lahart et al., 2019; Papale et al., 2025). All these benefits could be due to feelings of connectedness with nature and visual recognition of characteristic features such as the color green and geometrical fractals (Joye and van den Berg, 2011; Akers et al., 2012; Cervinka et al., 2012; Lahart et al., 2019). The present findings could suggest that these benefits observed in natural outdoor environments may be replicated in virtual settings. Indeed, the use of virtual natural environments can, at least in part, reproduce the affective mechanisms observed in green outdoor activities, such as enhanced positive feelings and increased engagement. The Autotelic Experience findings are conceptually consistent with PACES findings, where participants achieved higher scores during both VR conditions compared to No VR cycling. Although the association between these measures was not tested in the present study, the consistency between the Autotelic Experience and enjoyment results could indicate that both conditions are intrinsically rewarding and enjoyable. Enjoyment is both a predictor and outcome of physical activity participation (Williams et al., 2006; Dacey et al., 2008; Mullen et al., 2011). Expected enjoyment from physical activities can increase exercise intentions, and the mere anticipation of positive emotions predicts physical activity adoption and maintenance (Mullen et al., 2011; Ruby et al., 2011). Therefore, although the natural VR scene could have slight benefits on flow experience and engagement during cycling exercise, both VR environments enhanced the pleasurable aspects of cycling, transforming physical activity into a more positive and enjoyable experience, as suggested by previous studies (Polechoński et al., 2024; Poli et al., 2024).

This study provides preliminary insights into the benefits of VR on cycling exercise, particularly regarding the choice of scenario that best reflects psychological parameters. The findings indicate that the use of non-immersive VR can enhance perceptual responses, such as enjoyment and some dimensions of flow, contributing to a more positive and engaging exercise experience, especially when participants use a natural environment. Exercising in VR with nature could be used as an effective tool to promote exercise adherence and well-being, particularly among individuals who may find indoor cycling training monotonous or less motivating. However, it is important to recognize some limitations. Only three flow dimensions, Action-Awareness Merging, Loss of Self-Consciousness, and Unambiguous Feedback, showed higher scores compared with the No VR condition, while no significant differences were found between the Urban VR and No VR conditions. Therefore, it cannot be stated that the nature-based virtual environment provides a uniquely higher level of engagement than the urban VR setting. Rather, the observed effects may suggest that using a natural VR scenario can improve some aspects of psychological immersion than traditional indoor cycling without VR. Individual preference and perceived relevance of the virtual environment may also have influenced flow, as engagement is likely enhanced when scenes align with personal interests, familiarity, or intrinsic enjoyment. Such alignment may promote more positive affective responses and autonomous motivation, which have been consistently identified as key psychological determinants of physical activity behavior (Cortis et al., 2017). Environmental familiarity could represent a potential limitation. In fact, the natural environment (Palmer Creek Gravel Road) was likely less familiar to United Kingdom participants than the urban condition (London), and novelty effects may have influenced responses during the sessions. Moreover, the cycling exercise protocol was short (10 min), which may have limited the change in flow responses. Longer exercise sessions might provide an increase or decrease in these effects, allowing a realistic representation of a typical training session. The sample consisted of young physically active adults, which may limit the generalizability of the findings to other populations, such as older adults or sedentary individuals. Moreover, physically active participants may have exhibited higher intrinsic motivation toward exercise, which may have contributed to high scores on the FSS dimensions during VR conditions. Lastly, although the order of conditions was randomized, both VR scenarios were performed on the same day, which may have introduced fatigue effects despite the 30-min recovery period.

Future research should explore the long-term effects of different VR environments on adherence, as well as on exercise and psychological responses during short and long exercise sessions or with different intensities. Extending the present study to populations that are not currently meeting physical activity guidelines, such as sedentary individuals or older adults, may be particularly relevant to evaluate the potential of VR-based exercise as a strategy to promote engagement in physical activity. Moreover, the familiarity of the virtual environment (such as local scenes) could be further examined. It could also be interesting to examine whether immersive VR (i.e., head-mounted displays) exhibits greater exercise experience and enjoyment than non-immersive setups, as well as to incorporate neurophysiological measures to evaluate the mechanisms behind the benefits of different VR scenarios. Lastly, future studies could evaluate the association between flow dimension, enjoyment, and other psychological measures in different VR scenarios, to better understand the mechanisms underlying positive exercise experiences and their potential role in promoting exercise adherence.

## Data Availability

The raw data supporting the conclusions of this article will be made available by the corresponding author, without undue reservation.
